# Effect of acupuncture on cerebrovascular reserve in patients with acute cerebral infarction: protocol for a randomized controlled pilot study

**DOI:** 10.1186/s13063-017-2013-5

**Published:** 2017-06-24

**Authors:** Shaosong Wang, Tingting Ma, Linpeng Wang, Lu Liu, Huilin Liu, Bin Li, Yuanbo Fu

**Affiliations:** Acupuncture and Moxibustion Department, Beijing Hospital of Traditional Chinese Medicine Affiliated to Capital Medical University, Backstreet Gallery No. 23, Dongcheng District, Beijing, China

**Keywords:** Stroke, Cerebrovascular reserve, Acupuncture, Acute cerebral infarction, Acupoints

## Abstract

**Background:**

The incidence of cerebral infarction has been growing year by year in China and around the world. According to clinical observation, acupuncture utilizing the “waking up the spirit” needling method is widely used in patients with cerebral infarction, though the underlying mechanisms remain unclear. Additionally, a number of studies have begun to focus on the relationship between cerebrovascular reserve (CVR) and cerebral infarction. The present study aims to investigate whether CVR is one of potential mechanisms underpinning this effect of acupuncture on patients with cerebral infarction.

**Methods:**

This is a single-centre, prospective, single-blinded, randomized controlled pilot study. Sixty eligible patients will be randomized into an intervention group (waking up the spirit acupuncture) and a control group (hand and foot 12-meridian acupuncture) in a 1:1 ratio. All treatments will be conducted once a day on weekdays followed by a 2-day rest period on the weekend, over a total treatment course of 2 weeks. The primary outcome measures are cerebrovascular reserve (CVR) capacity and Breath-holding Index (BHI) which will be evaluated at baseline and 2 weeks after the first acupuncture treatment, and the secondary outcome measures are National Institutes of Health Stroke Scale (NIHSS) and Barthel Index scores which will be used to further evaluate the efficacy of the intervention.

**Discussion:**

Cerebrovascular reserve is an independent risk factor for the occurrence, progression, and recurrence of cerebral infarction that requires attention. This trial aims to investigate whether acupuncture utilizing the waking up the spirit needling method can improve CVR capacity in patients with acute cerebral infarction, thus reducing NIHSS scores and preventing further progression of the disease. Furthermore, data and evidence gained from this study will be utilized in the development of future research projects regarding the effects of acupuncture in patients with acute cerebral infarction.

**Trial registration:**

ISRCTN, ID: ISRCTN99117074. Registered on 20 April 2016.

**Electronic supplementary material:**

The online version of this article (doi:10.1186/s13063-017-2013-5) contains supplementary material, which is available to authorized users.

## Background

Acute cerebral infarction is a relatively common condition whose incidence is increasing around the world, with high rates of morbidity, mortality, and disability [1–6]. Increasingly stressful social conditions and unhealthy lifestyles contribute to the increased incidence of cerebral infarction in younger individuals [7–10], negatively impacting both families and society at large [2, 3, 11]. The incidence of cerebral infarction in China is nearly the highest in the world, and cerebrovascular disease has ranked among the leading causes of death in China since 2008 [2, 12, 13] and has become the third leading cause of death, behind malignant neoplasms and heart disease, according to a 2014 fact sheet published by the Chinese CDC [14]. Systematic analyses have estimated that about 1.7 billion Chinese patients die of cerebral infarction every year, with a recurrence rate greater than 17.1% [11] in surviving individuals.

Cerebral infarction is closely related to cerebrovascular stenosis or cerebral thrombosis. Deficiencies in cerebral perfusion are associated with three compensatory mechanisms of the cerebrovasculature: establishment of collateral circulation, cerebrovascular reserve (CVR), and metabolic reserve. CVR—also known as cerebral blood flow reserve, cerebral perfusion reserve, and cerebral circulation reserve—refers to the ability of the cerebrovasculature to maintain normal cerebral blood flow under physiological or pathological stimulation by via compensatory adjustment of small arteries and capillaries [15]. In the early stages of cerebral ischemia, collateral circulation is established, and blood flow velocity is increased in order to maintain cerebral perfusion volume. When the collateral circulation can no longer compensate for the deficiency in perfusion, CVR begins to increase perfusion by dilating small cerebral arteries and capillaries. When CVR can no longer support further increases in cerebral perfusion volume, brain tissue can only maintain the intracranial blood supply by increasing oxygen fraction extraction. However, as the degree of cerebral anoxia worsens, acute infarction becomes more likely to occur [16]. Patients with low CVR tend to experience more severe hypoperfusion, ischemia, and hypoxia, leading to infarction and further tissue damage [17]. Deficient CVR is not only an independent risk factor for cerebral infarction [18–21] but also a risk factor for the progression [22–25] and recurrence [26] of cerebral infarction. Therefore, the development of novel strategies for increasing CVR in patients with acute infarction is crucial for preventing the progression and recurrence of infarction.

The treatment of acute ischemic cerebral infarction aims to maintain the blood supply and oxygenation of the ischemic penumbra. CVR is closely connected with cerebrovascular diseases and research on cerebrovascular disease has become increasingly focused on CVR [27–32]. Research has also indicated that acupuncture exerts specific effects on blood flow velocity, vascular reactivity, and circulation [33–36], suggesting that acupuncture may improve outcomes in patients with diminished CVR. To the best of our knowledge, however, reports have yet to demonstrate the effect of acupuncture on CVR. Therefore, the present study aims to investigate whether acupuncture utilizing [37] the “waking up the spirit” needling method can improve CVR capacity in patients with acute cerebral infarction. Different methods of acupuncture have varying influences on blood flow, and stronger stimulation may be required to improve blood flow in patients with cerebral infarction [38–41]. Acupuncture utilizing the waking up the spirit needling method provides intense stimulation of the head and limbs by means of bloodletting at certain acupoints, and the positive effects of acupuncture in general have been documented with regard to a number of conditions, including increased cerebral blood flow in the region surrounding the ischemic area [42–44]. Butylphthalide [45] has an effect on patients with acute cerebral infarction; the underlying mechanism maybe that it changes CVR by changing blood flow. As with butylphthalide, we are trying to explore whether acupuncture, utilizing the waking up the spirit needling method, has the same routine mechanism that can improve CVR in patients with acute cerebral infarction, thus reducing scores on the National Institutes of Health Stroke Scale (NIHSS) and preventing further disease progression.

Though a variety of methods may be used to assess CVR (positron emission tomography (PET), single photon emission computed tomography, computed tomography (CT) angiography, magnetic resonance (MR) angiography, transcranial Doppler (TCD) sonography, etc. [46], PET is the most effective in evaluating the capacity for vascular dilatation. However, this method is expensive and relatively underutilized in China. TCD, on the other hand, allows for the noninvasive examination of local blood flow velocity in the middle cerebral artery [47]. Blood flow is proportional to blood flow velocity [48], allowing for the indirect evaluation of CVR using TCD. TCD has been utilized in clinical examination for nearly 30 years and is easy to perform, noninvasive, inexpensive, and repeatable [47]. Some studies have also reported that TCD is effective in evaluating cerebrovascular reactivity using the breath-holding test. [49–51]. For these reasons, we chose TCD to assess CVR in the present study.

## Methods

### Objectives

The present study aims to test the hypothesis that acupuncture utilizing the waking up the spirit needling method can improve CVR capacity in patients with acute cerebral infarction, thus reducing NIHSS scores and preventing further disease progression.

### Recruitment

**Fig. 1:**
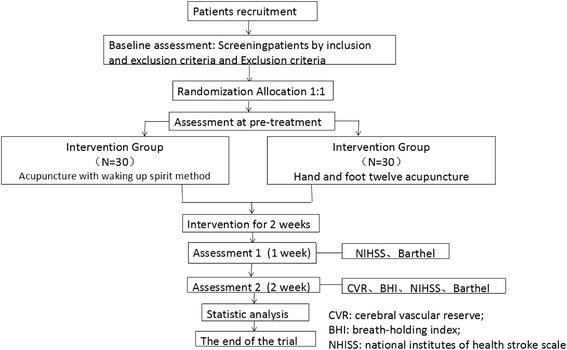
Flow chart

**Fig. 2:**
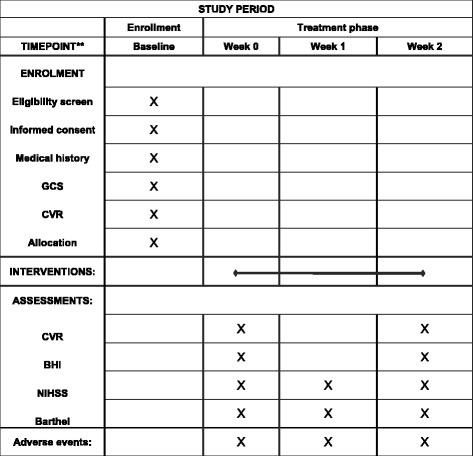
Standard Protocol Items: Recommendations for Interventional Trials (SPIRIT) Figure

The present study is a single-center, prospective single-blinded, randomized controlled pilot trial to be carried out in three stages (Fig. 1): a recruitment period prior to baseline assessment and randomization, a treatment and observation period of 2 weeks, and a final follow-up period. All participants will be recruited from the three acupuncture wards of Beijing Hospital of Traditional Chinese Medicine at Capital Medical University (Beijing, China). In the first stage, we will seek out potentially qualified patients according to the inclusion and exclusion criteria and provide them with detailed information about the study such as the research objective, study procedure, and both the potential benefits and risks. If a patient agrees to participate, they will be asked to voluntarily provide written informed consent, following which baseline assessments and randomization will be conducted. Table 1 depicts the timetable of the trial. Figure 2 illustrates the Standard Protocol Items: Recommendations for Interventional Trials (SPIRIT) Figure which the trial protocol follows (Additional file 1).

**Table 1:** Time table

	Baseline (before treatment)	Treatment			Follow-up and data collection
		Week 0	Week 1	Week 2	Data collection and analysis
Patients entered					
Medical history	√				
GCS	√				
CVR	√				
Sign the informed consent	√				
Randomization	√				
Intervention		√	√	√	
Outcomes					
Primary outcomes					
CVR		√		√	
BHI		√		√	
Secondary outcome					
NIHSS		√	√	√	
Barthel Index		√	√	√	
Adverse events	√	√	√	√	√
Data collection					√

*GCS* Glasgow Coma Scale, *CVR* cerebrovascular reserve, *BHI* Breath-holding Index, *NIHSS* National Institutes of Health Stroke Scale

### Design

#### Randomization and allocation concealment

The randomization scheme was provided by a random number table. A set of 60 random numbers was generated by a designated computer, and each number was then divided by 2. Resulting numbers with a remainder of 0 corresponded to the treatment group, while those with a remainder of 1 corresponded to the control group. However, this system resulted in 36 control numbers and only 24 treatment numbers, so it was necessary to re-assign six numbers from the control group to the treatment group. The next six numbers were chosen from the random number table and divided by 36, resulting in the following remainders: 26, 24, 6, 2, 22, and 14. Therefore, we assigned patient numbers 26, 24, 6, 2, 22, and 14 into the treatment group, resulting in a total of 30 patients in each group. Participants will be allocated into one of these two groups according to the order in which they are recruited.

#### Blinding

The therapists will be aware of the treatments in both groups as they are experienced enough to distinguish between the treatment methods; however, the participants will remain blinded to the treatment. Participants will be told that they have been randomly allocated to either the treatment group or the control group, and they will be treated with bloodletting using a three-edged needle, regular acupuncture, or both, based upon their condition. Moreover, the study will utilize independent data managers and statisticians who will also remained blinded to the intervention methods throughout the trial. All therapists, data managers, and statisticians have been requested to refrain from communication with each other regarding the study.

### Participants

#### Sample size

As we were unable to find any publication regarding acupuncture for CVR capacity, we could not calculate the appropriate sample size according to the sample size calculation formula. We determined an appropriate sample size of 60 for a pilot study. Both the treatment and control groups will contain 30 eligible participants. The outcomes of this study will aid in the calculation of the appropriate sample size for further randomized clinical trials.

#### Inclusion criteria

Patients of either sex are eligible for entry into this study if they meet the following inclusion criteria: (1) age between 30 and 80 years with acute cerebral infarction, with an onset time of less than 7 days, (2) confirmed as meeting the Western medicine diagnostic criteria for cerebral infarction issued by the American Heart Association/American Stroke Association (AHA/ASA) in 2015 [52], (3) confirmed as meeting the traditional Chinese medicine diagnostic criteria developed by the Stroke Diagnosis and Curative Effect Evaluation Standard (Draft) of the State Administration of Traditional Chinese Medicine, Acute Encephalopathy Research Group [53], (4) confirmation of diagnosis by head CT or MRI, (5) confirmed CVR impairment as assessed using the breath-holding-test, (6) NIHSS score within the range of 5–20 points, (7) Glasgow Coma Scale score ≥12 points, and (8) agreement to participate in this trial and signing an signed Informed Consent Form.

#### Exclusion criteria

Patients are ineligible if they meet the following exclusion criteria: (1) use of antithrombotics such as low molecular heparin for the treatment of another conditions, (2) carotid artery stent in situ, (3) severe heart, lung, or kidney disease, (4) inability to cooperate during ultrasound examination of the brain or quantitative evaluation due to another condition.

#### Discontinuation criteria

Reasons for discontinuation of treatment may include, but are not limited to, the following: (1) participant’s decision to discontinue treatment at any time for any reason, (2) investigator’s determination to discontinue treatment for the patient’s safety and best interests at any time, (3) inability to tolerate the treatment stimulation at any time during the course of the study, (4) occurrence of serious side effects during the treatment course, (5) exacerbation of the disease making it difficult for the participant to continue treatment, (6) inability of the participant to cooperate during assessment for any reason, and (7) concomitant therapy during the trial that may affect the study results.

#### Loss to follow-up

An intention-to-treat approach will be used, and reasons for which patients have been lost to follow-up must be recorded in detail and analyzed after the trial. If the number of cases lost to follow-up is within 10% of all participants, the data from these cases will not be included in the analysis. If the number of cases lost to follow-up exceeds 10%, data from the cases lost in the treatment group will be regarded as invalid, while data from the cases lost in the control group will be regarded as valid. Thus, the effective percentage of the treatment group will remain higher than that of the control group, allowing us to further analyze the efficacy of the intervention.

### Intervention

A bloodletting regimen, using a three-edged needle, will be performed based upon descriptions in the ancient literature and our clinical experience. The bloodletting will be performed by certified acupuncturists with at least 20 years of clinical experience. All treatment details will be standardized among practitioners who will receive relevant training and video guidance prior to the first acupuncture session. Patients will adopt a supine position during the treatment. The operators will sterilize the skin of an area of 3 cm around the acupoints with 75% alcohol. Participants in the treatment group will receive acupuncture utilizing the waking up the spirit needling method, while participants in the control group will be treated using 12-meridian hand and foot acupuncture. All treatments will be performed once a day on weekdays, followed by a 2-day rest period on weekends, for a total course of 2 weeks. Each treatment session will last approximately 35 min: the first 5 min will consist of the acupuncture procedure, following which the needles will be retained for 30 min in the patients of both the treatment and control groups.

#### Treatment group

Acupuncture utilizing the waking up the spirit needling method will be performed as follows. First, patients will adopt a supine position, then the operators will sterilize the skin of an area of 3 cm around acupoints with 75% alcohol. Acupuncture bloodletting will be conducted at acupoints *Bai Hui* (DU-20), *Si Shen Cong* (EX-HN1), and at 12 well-points using a three-edged needle which will be inserted into the skin at a depth of approximately 5–10 mm. The needle will be withdrawn immediately, and the skin around the pin hole will be squeezed to release a few drops of blood. The 12 well-points are *Shao Shang* (LU-11), *Shang Yang* (LI-1), *Li Dui* (ST-45), *Yin Bai* (SP-1), *Shao Chong* (HT-9), *Shao Ze* (SI-1), *Zhi Yin* (BL-67), *Yong Quan* (KI-1), *Zhong Chong* (PC-9), *Guan Chong* (SJ-1), *Zu Qiao Yin* (GB-44), and *Da Dun* (LR-1). Lastly, other points, such as *Ren Zhong* (GV-26), *Cheng Jiang* (CV-24), *Feng Chi* (GB-20), *He Gu* (LI-4), *Lao Gong* (PC-8), *Tai Chong* (LV-3), and *Yong Quan* (K-I1), will be chosen after the bloodletting, and the filiform needles will be inserted to depth of 10–15 mm and manually manipulated by rotation in order to produce a characteristic sensation known as “*De Qi*.” Techniques of mild reinforcing and attenuating will be applied, and the filiform needles will be retained for 30 min.

#### Control group

Twelve-meridian hand and foot acupuncture will be performed as follows. First, patients will be asked to adopt the supine position, and the operators will sterilize a 3-cm skin area around acupoints with 75% alcohol. Acupuncture including the *Qu Chi* (LI-11), *Nei Guan* (PC-6), *He Gu* (LI-4), *Yang Ling Quan* (GB-34), *Zu San Li* (ST-36), and *San Yin Jiao* (SP-6) points will be stimulated with filiform needles to a depth of 10–15 mm. The needles will be manually manipulated by rotation methods in order to produce a characteristic sensation known as *De Qi*. Techniques of mild reinforcing and attenuating will be applied, and the filiform needles will be retained for 30 min.

#### Follow-up

All participants will continue to undergo conventional therapy according to the National Clinical Guideline for Stroke [54]. Recurrence rate and Barthel Index scores will be assessed at the 3- and 6-month follow-ups for further information related to prognosis, though these will not be factored in to our assessment of the main outcomes.

### Outcome measures

#### Primary outcome measures: CVR and BHI

1. Cerebrovascular reserve (CVR)
  CVR will be calculated at baseline and 2 weeks after the first acupuncture session according to the following formula, in which the mean blood flow velocity (MFV) of the bilateral middle cerebral artery is measured using the transcranial Doppler (TCD) Breath-holding Test:
  $$ \begin{array}{l} CVR = \left( MFV\ \mathrm{after}\ \mathrm{breath}\hbox{-} \mathrm{holding}\ \hbox{--}\ MFV\ \mathrm{before}\ \mathrm{breath}\hbox{-} \mathrm{holding}\right)/ MFV\ \mathrm{before}\\ {}\ \mathrm{breath}\hbox{-} \mathrm{holding} \times 100\%.\end{array} $$
  CVR function is considered impaired if the calculated CVR is less than 20%.
  CVR will be accessed via the TCD breath-holding test. For this test, patients are instructed to practice holding their breath at least twice prior to assessment. Patients then adopt the supine position and are asked to breathe normally for 5 min. Two ultrasonic probes are then placed on the bilateral temporal window and connected to the EMS-9 W TCD machine (Nanjing Bangao Company, Nanjing, China). The ultrasonic probe is used to record at a depth of roughly 50–55 mm, measuring blood velocity in the bilateral middle cerebral artery (M1). Once an adequate signal has been obtained, the probes are fixed, and the MFV is recorded while the patient is in a calm state. The patient is then asked to hold their breath for at least 15 s, following which the MFV is re-recorded.
2. Breath-holding Index (BHI)
  BHI assessments will be conducted at baseline and 2 weeks after the first acupuncture session according to the following formula, in which the MFV of the bilateral middle cerebral artery is measured using the TCD breath-holding test:
  $$ \begin{array}{l} BHI = \left( MFV\ \mathrm{after}\ \mathrm{breath}\hbox{-} \mathrm{holding}\ \hbox{--}\ MFV\ \mathrm{before}\ \mathrm{breath}\hbox{-} \mathrm{holding}\right)/ MFV\ \mathrm{before}\ \\ {}\mathrm{breath}\hbox{-} \mathrm{holding} \times \left(100/\mathrm{time}\ \mathrm{holding}\ \mathrm{breath}\right).\end{array} $$

#### Secondary outcome measures: NIHSS and Barthel Index scores

1. NIHSS [55]
  Neurological function will be assessed using the National Institutes of Health Stroke Scale (NIHSS) at baseline, 1 week after the first acupuncture treatment, and 2 weeks after the first acupuncture treatment. The NIHSS is widely used for assessing neurological function defect symptoms.
2. Barthel Index [56]
  Functional ability will be assessed using the Barthel Index at baseline, 1 week after the first acupuncture treatment, and 2 weeks after the first acupuncture treatment.
  The Barthel Index is widely used for assessing quality of life of clinical patients.

#### Adverse events

Adverse events (AEs) are defined as negative or unintended clinical manifestations following the treatment [57]. Our investigators will collect information regarding AEs every 3 days. Participants will be instructed to report any abnormal reactions or uncomfortable feelings experienced to any researcher. All related and unexpected AEs will be recorded on Case Report Forms (CRFs) in detail, including time of occurrence, degree of AE, and possible causes. Patients with mild and moderate AEs will be treated for their symptoms and closely monitored as necessary by the researcher. Severe AEs will be reported to the Research Ethics Committee, which will provide medical advice to the research team within 48 h, and the Research Ethics Committee will determine whether the patient is eligible for further treatment associated with the study.

### Data management

All researchers including therapists, data entry clerks, the data collector, data manager, statistician, and outcome assessors will receive special training regarding the research contents and data management. During the recruitment period, our data collector will record the baseline characteristics of participants on CRFs, and all data will be assessed by the data manager. Then, as the study begins, the data collector will obtain CVR, BHI, NIHSS, and Barthel Index data at baseline and 2 weeks following the first treatment. Additional NIHSS and Barthel Index data will be obtained 1 week following the first treatment. The TCD assessment will be conducted by an appointed operator who has completed an advanced course of study in this field at Beijing Tiantan Hospital Affiliated with Capital Medical University.

Upon conclusion of the treatment period, all participant data will be completed and recorded on the original CRFs. The data will then be entered into Excel spreadsheets by two separate data entry clerks, following which the data manager will compare the accuracy of the two datasets. If any differences are noted, corrections will be made according to, and marked on, the original CRFs. The data will be managed in accordance with the Data Protection Act of 1998 [58]. All paper files related to the research will be saved in a locked filing cabinet, while electronic documents will be stored in a special computer which will remain password-protected and accessible only to the principal investigators. All research documents, including both the paper files and electronic documents, will be preserved for at least 5 years after publication. If readers have any questions regarding our published data, they will be permitted to contact our first author or corresponding author to ask for the original data.

### Statistical analysis

Data analysis will be performed by statisticians who are blinded to the entire allocation and intervention process. The statisticians are affiliated with the Research Center of Clinical Epidemiology at Peking University Third Hospital in Beijing, China, which is one of the most authoritative statistics centers in the country. An intention-to-treat analysis will be conducted using the SPSS statistical software package (V.22.0) (International Business Machines Corporation). Two-tailed analyses will be conducted, with the level of statistical significance defined as *P* < 0.05. Baseline characteristics, such as gender, age, NIHSS score, GCS score, previous duration, and impaired CVR, will be analyzed. The categorical data will be described as *n* (%), and continuous data using mean ± SD. If the data meets the standard of the parameter test, a chi-square test will then be conducted. Independent sample *t* tests will be used for comparisons among the groups, while paired *t* tests will be used for within-group comparisons. The efficacy of the intervention will be compared between the two groups using *χ* ^2^ analysis.

### Ethical considerations

The trial protocol is in accordance with the principles of the Declaration of Helsinki [59] and was approved by the Research Ethical Committee of Beijing Hospital of Traditional Chinese Medicine at Capital Medical University on 30 November 2015 (Ethics Reference Number: 20151130). This trial was registered at ISRCTN (ID: 99117074). Each participant will be notified regarding the study protocol. Written informed consent will be obtained from every participant.

## Discussion

This trial aims to investigate whether acupuncture utilizing the waking up the spirit needling method can improve CVR capacity in patients with acute cerebral infarction, thus reducing NIHSS scores and preventing further disease progression. Some studies [60–62] have demonstrated that acupuncture may improve symptoms in patients with cerebral infarction and, according to clinical observations, acupuncture utilizing the waking up the spirit needling method may produce similar effects. Furthermore, increased focus has been directed at the relationship between CVR and cerebral infarction [32]. Acupuncture is widely utilized in the treatment of cerebral infarction, though the mechanisms underlying its effects remain unclear. In this randomized, controlled study we expect that participants will experience pain relief as well as improved function and strength. Furthermore, data and evidence gained from this study will be utilized in the development of future research projects regarding the effects of acupuncture in patients with acute cerebral infarction.

The risks of taking part in this trial are minimal. Acupuncture is a very safe treatment when conducted by properly trained clinicians. Occasionally, acupuncture can make people feel nauseous, faint, or experience a temporary increase in pain either during or after treatment.

The global outlook for cerebral infarction is not good [4, 63], and both first- and second-level prevention are critical at reducing the impact of this potentially life-threatening and devastating condition [63–70]. CVR is an independent risk factor for the occurrence [18–21], progression [22–25], and recurrence [26] of cerebral infarction that requires attention. Some researchers have even proposed that CVR should be assessed regularly, as one of the key targets for prevention [71].

Acupuncture provides distinct advantages in that it is easy, convenient, economic, and effective. The waking up the spirit needling method and the hand and foot 12-meridian needle method are commonly used in China for the treatment of stroke patients. Both of them have shown curative effect in clinical practice. It is noted, however, that the waking up the spirit needling method using bloodletting with a three-edged needle, provides stronger stimuli to the head than the hand and foot 12-meridian needle method. Since those bloodletting acupoints are located on the head, it is reasonable to hypothesize that this method might exert an impact on cerebral blood flow. The same effect is unlikely to be obtained by the hand and foot 12-meridian needle method which takes acupoints from the extremities. Significant differences in blood flow have been observed between strong stimulation and regular acupuncture, though the mechanism underlying these differences remain unclear [38–41]. Butylphthalide [45] has an effect on patients with acute cerebral infarction, the underlying mechanism maybe that it changes the CVR by changing blood flow. As with butylphthalide, we hypothesize that acupuncture utilizing the waking up the spirit needling method can improve CVR capacity in patients with acute cerebral infarction.

We chose the hand and foot 12-meridian needle method as the control intervention. The 12 acupoints stimulated in this method are located on the arms and legs, and the stimulation provided by this technique is considered to be less than that of bloodletting utilizing a three-edged needle. Therefore, the procedure should have less effect on CVR and cerebral blood flow. The 12-acupoint method is also taught by masters of acupuncture and is quite popular in clinical treatment. Furthermore, research has indicated that it is effective in improving patients’ symptoms [72]. Therefore, it is a reasonable control intervention that meets ethical standards.

To our knowledge, the present trial marks the first attempt to investigate the efficacy of acupuncture with regard to improving CVR. The results of this trial may help to elucidate the mechanism underlying the effects of acupuncture in patients with acute stroke, which may significantly aid in the development of clinical treatment protocols and further studies regarding the underlying mechanisms of these effects. As research has indicated that adequate CVR is crucial for preventing the occurrence, progression, and recurrence of cerebral infarction, the results of this trial may also highlight the importance of regular assessment of CVR.

### Trial status

At the time of manuscript submission, we started to seek out potentially appropriate patients from the three acupuncture wards of Beijing Hospital of Traditional Chinese Medicine Affiliated to Capital Medical University according to the inclusion and exclusion criteria and provided them with detailed information about the study. About six patient agreed to participate, after which followed written informed consent and baseline assessments and randomization. We also tried to do some test work before the real recruitment started. However, by the time of manuscript submission we had not started to apply the interventions.

## Additional file

Additional file 1: SPIRIT Checklist. The SPIRIT Checklist document. (PDF 217 kb)

## Abbreviations

AE: Adverse event
AHA: American Heart Association
ASA: American Stroke Association
BHI: Breath-holding Index
CRF: Case Report Form
CT: Computed tomography
CVR: Cerebrovascular reserve
GCS: Glasgow Coma Scale
M1: Middle cerebral artery
MFV: Mean blood flow velocity
MR: Magnetic resonance
NIHSS: National Institutes of Health Stroke Scale
PET: Positron emission tomography
TCD: Transcranial Doppler
