# Supplementary material for: Effect of acupuncture on cerebrovascular reserve in patients with acute cerebral infarction: protocol for a randomized controlled pilot study
Source: Trials. 2017 Jun 24;18:292. doi: 10.1186/s13063-017-2013-5 (PMC5482967; doi:10.1186/s13063-017-2013-5)
Supplement: Additional file 1: — SPIRIT Checklist. The SPIRIT Checklist document. (PDF 217 kb) [file 13063_2017_2013_MOESM1_ESM.pdf]

SPIRIT 2013 Checklist: Recommended items to address in a clinical trial protocol and related documents*

Section/item	Item No	Description	Addressed on page number
**Administrative information**			
Title	1	**Effect of acupuncture on cerebrovascular reserve in patients with acute cerebral infarction: study protocol for a randomized controlled trial**	______1_______
Trial registration	2a	**Trial registration number: ISRCTN99117074**	______3______
	2b		_____________
Protocol version	3	**2016-08-12 Version 1.0**	_____________
Funding	4	National Basic Research Program of China (Grant No. 2014CB543203), Beijing Natural Science Foundation (Grant No. 7154205), Beijing Municipal Administration of Hospitals Clinical Medicine Development of Special Funding Support (Grant No. ZYLX201412), Beijing Traditional Chinese Medicine Science and Technology Project (Grant No. JJ2014-10), and Beijing Natural Science Foundation (Grant No.7154206).	______21______

Roles and responsibilities	5a	S-SW, T-TM, and L-PW conceived and designed this study. S-SW and T-TM drafted and wrote the first manuscript describing the protocol. L-PW, LL, and H-LL revised the protocol. LL, H-LL, BL, and Y-BF participated in the design of the trial and will conduct the acupuncture sessions. All authors read and approved the final manuscript.	______21______
	5b	The Beijing Municipal Administration of Traditional Chinese Medicine (Telephone number: 0086 010 83970677)	______________
	5c	Beijing Municipal Administration of Traditional Chinese Medicine is the sponsor and will not affect the result of this trial.	______________
	5d	This trial will be monitored by the scientific research department of Beijing TCM Hospital.	______________
**Introduction**			
Background and rationale	6a	The incidence of cerebral infarction has been growing year by year in China and around the world. According to clinical observation, acupuncture utilizing the “waking up the spirit” needling method is widely used in patients with cerebral infarction, though the underlying mechanism remains unclear. Additionally, a number of studies have begun to focus on the relationship between cerebrovascular reserve and cerebral infarction. The present study aims to investigate whether acupuncture utilizing the waking up the spirit needling method can improve cerebrovascular reserve (CVR) capacity in patients with acute cerebral infarction, thus reducing scores on the National Institutes of Health Stroke Scale (NIHSS) and preventing further progression of the disease.	________1______

	6b	We chose the hand and foot twelve-meridian needle method as the control intervention. The twelve acupoints stimulated in this method are located on the arms and legs, and the stimulation provided by this technique is considered to be less than that of bloodletting utilizing a three-edged needle. Therefore, the procedure should have less effect on CVR and cerebral blood flow. The twelve acupoints method is also taught by masters of acupuncture and quite popular in clinical treatment. Furthermore, research has indicated that it is effective in improving patient symptoms. Therefore, it is a reasonable control intervention that meets ethical standards.	______19_______
Objectives	7	The present study aims to test the hypothesis that acupuncture utilizing the waking up the spirit needling method can improve CVR capacity in patients with acute cerebral infarction, thus reducing NIHSS scores and preventing further progression of the disease.	______6_______
Trial design	8	A randomized controlled trial	______________

Methods: Participants, interventions, and outcomes

Study setting	9	A single center in Beijing Hospital of Traditional Chinese Medicine	______________
Eligibility criteria	10	Patients of either sex are eligible for entry into this study if they meet the following inclusion criteria:(1) age between 30 and 80 years with acute cerebral infarction, with an onset time of less than 7 days; (2) confirmed to meet the Western medicine diagnostic criteria for cerebral infarction issued by the American Heart Association/American Stroke Association (AHA/ASA) in 2013[53]; (3) confirmed to meet the traditional Chinese medicine diagnostic criteria developed by the Stroke Diagnosis and Curative Effect Evaluation Standard (Draft) of the State Administration of Traditional Chinese Medicine, Acute Encephalopathy Research Group[54]; (4) confirmation of diagnosis by head CT or MRI; (5) confirmed CVR impairment as assessed using the breath-holding-test; (6) NIHSS score within the range of 5-20 points; （7）Glasgow Coma Scale ≥ 12 points; (8) agreement to participate in this trial and signed informed consent form.	________9______

Interventions	11a	A bloodletting regimen using a three-edged needle will be performed based upon descriptions in the ancient literature and our clinical experience. The bloodletting will be performed by certified acupuncturists with at least 20 years of clinical experience. All treatment details will be standardized among practitioners, who will receive relevant training and video guidance prior to the first acupuncture session. Participants in the treatment group will receive acupuncture utilizing the waking up the spirit needling method, while participants in the control group will be treated using twelve-meridian hand and foot acupuncture. All treatments will be performed once a day on weekdays, followed by a two-day rest period on weekends, for a total course of 2 weeks. Each treatment session will last approximately 35 minutes: The first 5 minutes will consist of the acupuncture procedure, following which the needles will be retained for 30 minutes in patients of both the treatment and control groups.	11
	11b	Reasons for discontinuation of treatment may include, but are not limited to, the following: (1) Participant's decision to discontinue treatment at any time for any reason; (2) investigator's determination to discontinue treatment for the patient's safety and best interests at any time; (3) inability to tolerate the treatment stimulation at any time during the course of the study; (4) occurrence of serious side effects during the treatment course; (5) exacerbation of the disease making it difficult for the participant to continue treatment; (6) inability of the participant to cooperate during assessment for any reason; (7) concomitant therapy during the trial that may affect the study results.	10
	11c	All participants will receive free treatment	
	11d	The interventions are based on conventional therapy according to the National Clinical Guideline for Stroke	
Outcomes	12	The primary outcome measures are cerebrovascular reserve (CVR) capacity and breath-holding index (BHI), which will be evaluated at baseline and 2 weeks after the first acupuncture treatment, and the secondary outcome measures are National Institutes of Health Stroke Scale (NIHSS) and Barthel Index scores, which will be used to further evaluate the efficacy of the intervention.	13
Participant timeline	13	Figure 2	

Sample size	14	As we were unable to find any publication regarding acupuncture for CVR capacity, we could not calculate the appropriate sample size according to the sample size calculation formula. Therefore, we referred to articles. regarding the improvement of CVR, used the sample size calculation formula: $N=Z^2\times(P\times(1-P)/E^2$, we made Z=1.96, E=5%, and P=0.935, we got the N=93.389296, and we also referred the usual proportion of patients with acute cerebral infarction in our acupuncture ward, from which we determined an appropriate sample size of 90.	______8_______
Recruitment	15	All participants will be recruited from the three acupuncture wards of Beijing Hospital of Traditional Chinese Medicine at Capital Medical University (Beijing, China).	______7_______

Methods: Assignment of interventions (for controlled trials)

Allocation:

Sequence generation	16a	The randomization scheme was provided by a random number table. A set of 90 random numbers was generated by a designated computer, and each number was then divided by two. Resulting numbers with a remainder of zero corresponded to the treatment group, while those with a remainder of 1 corresponded to the control group. However, this system resulted in 52 control numbers and only 38 treatment numbers, so it was necessary to re-assign seven numbers from the control group to the treatment group. The next seven numbers were chosen from the random number table and divided by 52, resulting in the following remainders: 26, 24, 6, 2, 22, 40, and 14. Therefore, we assigned patient numbers 26, 24, 6, 2, 22, 40, and 14 into the treatment group, resulting in a total of 45 patients in each group. Participants will be allocated into one of these two groups according to the order in which they are recruited.	________8______
Allocation concealment mechanism	16b		_____________
Implementation	16c		_____________

Blinding (masking)	17a	The therapists will be aware of the treatments in both groups, as they are experienced enough to distinguish between the treatment methods; however, the participants will remain blinded to the treatment. Participants will be told that they have been randomly allocated to either the treatment group or the control group, and they will be treated with bloodletting using a three-edged needle, regular acupuncture, or both based upon their condition. Moreover, the study will utilize independent data managers and statisticians who will also remained blinded to the intervention methods throughout the trial. All therapists, data managers, and statisticians have been informed to refrain from communication with each other regarding the study.	_______8______
	17b	-	_____________

Methods: Data collection, management, and analysis

Data collection methods	18a	All researchers including therapists, data entry clerks, the data collector, data manager, statistician, and outcome assessors will receive special training regarding the research contents and data management. During the recruitment period, our data collector will record the baseline characteristics of participants on CRFs, and all data will be assessed by the data manager. Then, as the study begins, the data collector will obtain CVR, BHI, NIHSS, and Barthel Index data at baseline and 2 weeks following the first treatment. Additional NIHSS and Barthel Index data will be obtained 1 week following the first treatment.	______15_______
	18b	Dropouts and withdrawals from the study will be recorded in detail.	_____________
Data management	19	Upon conclusion of the treatment period, all participant data will be completed and recorded on the original CRFs. The data will then be entered into Excel spreadsheets by two separate data entry clerks, following which the data manager will compare the accuracy of the two datasets. If any differences are noted, corrections will be made according to and marked on the original CRFs. The data will be managed in accordance with the Data Protection Act of 1998. All paper files related to the research will be saved in a locked filing cabinet, while electronic documents will be stored in a special computer, which will remain password-protected and accessible only to the principal investigators. All research documents, including both the paper files and electronic documents, will be preserved for at least 5 years after publication. If readers have any questions regarding our published data, they will be permitted to contact our first author or corresponding author to ask for the original data.	_______16______

Statistical methods	20a	Data analysis will be performed by statisticians who are blinded to the entire allocation and intervention process. The statisticians are affiliated with the Research Center of Clinical Epidemiology at Peking University Third Hospital in Beijing, China, which is one of the most authoritative statistics centers in the country. An intention-to-treat analysis will be conducted using the SPSS statistical software package (V.22.0)(International Business Machines Corporation). Two-tailed analyses will be conducted, with the level of statistical significance defined as $P < 0.05$. Baseline characteristics such as gender, age, NIHSS score, GCS score, previous duration, and impaired CVR will be analyzed. The categorical data will be described as n (%), and continuous data using mean±SD. If the data meets the standard of the parameter test, a chi-square test will then be conducted. Independent sample t-tests will be used for comparisons among the groups, while paired t-tests will be used for within-group comparisons. The efficacy of the intervention will be compared between the two groups using $\chi 2$ analysis.	_____16,17_____
	20b	-	______________
	20c	An intention-to-treat approach will be used, and reasons for which patients have been lost to follow up must be recorded in detail and analyzed after the trial. If the number of cases lost to follow up is within 10% of all participants, the data from these cases will not be included in the analysis. If the number of cases lost to follow up exceeds 10%, data from the cases lost in the treatment group will be regarded as invalid, while data from the cases lost in the control group will be regarded as valid. Thus, the effective percentage of the treatment group will remain higher than that of the control group, allowing us to further analyze the efficacy of the intervention.	____10,11______

Methods: Monitoring

Data monitoring	21a	This trial will be monitored by the scientific research department of Beijing TCM Hospital.	______________
	21b	-	______________

Harms	22	Adverse events (AEs) are defined as negative or unintended clinical manifestations following the treatment. Our investigators will collect information regarding adverse events every three days. Participants will be instructed to report any abnormal reactions or uncomfortable feelings experienced to any researcher. All related and unexpected AEs will be recorded on Case Report Forms (CRFs) in detail, including time of occurrence, degree of AE, and possible causes. Patients with mild and moderate AEs will be treated for their symptoms and closely monitored as necessary by the researcher. Severe AEs will be reported to the Research Ethics Committee, which will provide medical advice to the research team within 48 h, and the Research Ethics Committee will determine whether the patient is eligible for further treatment associated with the study.	______15_______
Auditing	23	The study will be audited by Beijing Municipal Administration of Traditional Chinese Medicine every year.	_____________
**Ethics and dissemination**			
Research ethics approval	24	Ethical approval was granted on February 3, 2016, by the Research Ethical Committee of Beijing Hospital of Traditional Chinese Medicine Affiliated with Capital Medical University (ref: 20151130).	______20_______
Protocol amendments	25	If it is nessrary to modify the protocol, we should submit applications to Beijing Municipal Administration of Traditional Chinese Medicine	_____________
Consent or assent	26a	Researchers from will obtain informed consent or assent from potential trial participants. Participants will write informed consent.	_____________
	26b	-	_____________
Confidentiality	27	Research data will be gathered and saved. Paper files will be kept in a locked filing cabinet. Electronic documents will be stored in a password protected computer, with access restricted to the principal investigator. All research documents will be preserved for at least 5 years after publication.	_____________
Declaration of interests	28	None declared competing interests	______20_______
Access to data	29	Dataset will be stored in a computer which will be password protected, with access restricted to the principal investigator	_____________
Ancillary and post-trial care	30	-	_____________

Dissemination policy	31a	The results will be published after the study.	______________
	31b	Researcers in this trial will have authorship eligibility.	______________
	31c	The results will be published.	______________
**Appendices**			
Informed consent materials	32	Written informed consent will be obtained from all participants.	______________
Biological specimens	33		______________

*It is strongly recommended that this checklist be read in conjunction with the SPIRIT 2013 Explanation & Elaboration for important clarification on the items. Amendments to the protocol should be tracked and dated. The SPIRIT checklist is copyrighted by the SPIRIT Group under the Creative Commons "Attribution-NonCommercial-NoDerivs 3.0 Unported" license.
